# Invariant texture perception is harder with synthetic textures: Implications for models of texture processing

**DOI:** 10.1016/j.visres.2015.01.022

**Published:** 2015-02-07

**Authors:** Benjamin Balas, Catherine Conlin

**Affiliations:** Department of Psychology, Center for Visual and Cognitive Neuroscience, North Dakota State University, Fargo, ND USA; Department of Psychology, North Dakota State University, Fargo, ND USA

**Keywords:** Texture perception, summary statistics, invariance

## Abstract

Texture synthesis models have become a popular tool for studying the representations supporting texture processing in human vision. In particular, the summary statistics implemented in the Portilla-Simoncelli (PS) model support high-quality synthesis of natural textures, account for performance in crowding and search tasks, and may account for the response properties of V2 neurons. We chose to investigate whether or not these summary statistics are also sufficient to support texture discrimination in a task that required illumination invariance. Our observers performed a match-to-sample task using natural textures photographed with either diffuse overhead lighting or lighting from the side. Following a briefly presented sample texture, participants identified which of two test images depicted the same texture. In the *illumination change* condition, illumination differed between the sample and the matching test image. In the *no change* condition, sample textures and matching test images were identical. Critically, we generated synthetic versions of these images using the P-S model and also tested participants with these. If the statistics in the P-S model are sufficient for invariant texture perception, performance with synthetic images should not differ from performance in the original task. Instead, we found a significant cost of applying texture synthesis in both lighting conditions. We also observed this effect when power-spectra were matched across images (Experiment 2) and when sample and test images were drawn from unique locations in the parent textures to minimize the contribution of image-based processing (Experiment 3). Invariant texture processing thus depends upon measurements not implemented in the P-S algorithm.

## Introduction

Natural visual stimuli can vary substantially in appearance as a function of illumination conditions, the observer's distance to the stimulus, viewpoint or pose relative to the observer, and planar rotation. Indeed, a single real stimulus out in the world (e.g an object or texture) may present infinite variations in 2D appearance depending on the specific viewing conditions. Nonetheless, observers are typically able to cope with appearance variation reasonably well, achieving useful (if limited) levels of perceptual constancy with complex stimuli like familiar faces (Burton et al., 1999), real and nonce objects (Bulthoff & Edelman, 1992), and scenes (Xiao et al., 2010).

Texture and material perception both also exhibit invariance to ecologically-relevant changes in appearance to some extent. Observers can typically recognize or match textures across changes in planar rotation, changes in scale, and changes in illumination and can rapidly categorize images of materials taken in unconstrained settings (Sharan, L., Rosenholtz, R. & Adelson, 2009; Wiebel, Valsecchi & Gegenfurter, 2013). Also, as Fleming (2014) observes for material perception, the sheer range of diverse images that can reliably be labeled as “plastic” or “metal”, for example, suggests that the human visual system has some impressive means of compensating for variation in a range of parameters and extracting robust estimates of properties that are relevant to material categorization. To some extent, observers' ability to be both selective about what images they assign a material category (e.g. “glossy” to and generalize the same category to a diverse set of appearances suggests that some simple features that are useful tools for material perception in some settings (e.g., skewness of the pixel intensity histogram; Motoyoshi et al., 2007; Sharan et al., 2008) are likely not the sole basis for material inference (Anderson & Kim, 2008). Also, texture and material constancy is not perfect – Ho, Landy, & Maloney (2006) demonstrated for example, that roughness judgments regarding artificial textures were affected by illumination, a result that suggests that the measurements used to characterize illumination and roughness may to some extent be confounded by the visual system. Variation in viewpoint also appear to affect roughness judgments in a similar fashion (Ho, Maloney & Landy, 2007), which may suggest that whatever degree of perceptual constancy the visual system is able to achieve for textures may be constrained by some set of features (what Ho et al. refer to as *pseudocues*) that do not provide perfect information for invariant recognition.

Are there specific computational features that may be reasonable for achieving invariant texture recognition (and explain the various failures of perceptual constancy that have been observed)? The fact that textures (and materials) do not have consistent shape rules out some large classes of visual features that are useful in other domains. For example, hierarchical models of invariant object recognition (e.g. HMAX, Riesenhuber & Poggio, 1999) are likely to be most useful for recognizing objects with well-defined shape and consistent relationships between local and global contours, conditions that textures and materials typically do not meet. For texture recognition and discrimination, representations of visual structure that are less position-dependent are likely to be better. A useful shorthand for such representations is *summary statistics*, by which we refer to a broad class of image measurements that describe appearance using visual features considered in the aggregate: histograms of filter ouputs, correlation functions between wavelet coefficients, or moments of intensity histograms. Summary statistics are in general useful for texture encoding because they inherently reflect some basic properties of texture perception. For example, they are naturally invariant to simple transformations of texture appearance like translation. Nonetheless, it remains far from clear what specific summary statistics the visual system may use for texture and material perception in general. A range of different feature vocabularies have been proposed to account for human performance with different kinds of textures and different tasks, including the “needle” statistics proposed by Julesz (1981), center-surround filter outputs (Bergen & Adelson, 1988), and the “back-pocket” model of texture perception (Landy & Graham, 2004). To our knowledge, the specific problem of how invariant texture recognition is achieved has received comparatively little attention – while a number of computational models that purport to achieve some level of invariant texture recognition have been developed (Varma & Zisserman, 2002; 2009; Xu, Ji, & Fermuller, 2009), we are not aware of any work with human observers exploring candidate features that the human visual system may use to recognize and discriminant natural textures in an invariant fashion.

In the present study, we examine the extent to which a specific set of summary statistics, those implemented by the Portilla-Simoncelli (P-S) texture synthesis algorithm, support matching of texture samples and texture properties given changes in illumination. This is a useful candidate model to consider for a number of reasons: First, compared to other parametric models of texture appearance, the P-S algorithm reliably generates high quality synthetic images for a wide range of natural textures. Second, the P-S model has been used in prior behavioral work (Balas, 2006; 2012) to demonstrate that the features used as the basis of the model have some perceptual validity. Finally, the model has also been used in recent years both as a model of peripheral vision in general (Rosenholtz, 2000) and to account for the properties of cells in the ventral visual stream that may process summary statistics of appearance (Freeman & Simoncelli, 2011). Taken together, these various lines of research suggest that the P-S algorithm is a particularly good target model for investigating the extent to which invariant texture perception may be supported by summary statistics. Here, we do this by implementing a texture discrimination task designed to reveal the extent to which the P-S representation of texture appearance is sufficient for texture recognition given naturalistic appearance variation.

We asked observers to perform a match-to-sample task that required them to match sample and test textures across changes in illumination using both original and synthetic versions of the stimuli. Similar to (though see below) the logic employed in recent studies that used “mongrels' of peripherally-viewed stimuli (Balas, Nakano & Rosenholtz, 2009; Rosenholtz et al., 2011), we assumed that if the P-S algorithm is indeed a sufficient appearance code for invariant texture perception, performing our task with synthetic images should be no more challenging than with original images. Should this be the case, it would support the claim that the summary statistics in the model allow the visual system to cope with changes in illumination by constraining the appearance of images of the same texture under different lighting conditions to be more similar to one another than images of different textures. If, however, we observe a significant cost when observers complete our task using synthetic images, it would suggest that the summary statistics used in the P-S model do not offer a sufficient code for invariant texture processing. This result would suggest that invariant texture recognition may depend on higher-order statistics than those contained in the P-S model, or possibly even that summary statistics in general may not be an adequate tool for invariant texture perception.

An important caveat to our use of synthetic images here is that this study is not an examination of the role of “mongrels” and summary statistics in peripheral vision. While it would certainly be interesting to examine the capabilities of peripheral vision with regard to invariant texture recognition, in the current manuscript we are not characterizing the properties of peripheral vision nor using the P-S model as a proxy for computations that may occur in the periphery. Instead, we are examining the extent to which this particular model of texture appearance carries sufficient information about texture appearance for observers to achieve some level of invariant texture processing. Thus, while this study and prior work with “mongrels” share some qualities (the use of P-S textures) the goals of the current study are distinct and we do not comment here on how these tasks might play out in the visual periphery. Instead, in three complementary experiments, we offer insights into what features do and do not appear to make contributions to observers' ability to achieve invariant texture matching. In Experiment 1, we examine observers' ability to match original and synthetic texture samples subject to either changing illumination or stable illumination. In Experiment 2, we examine the contribution of luminance and contrast to this problem domain by imposing matched power spectra on our test images. Finally, in Experiment 3, we examine invariant texture processing by asking observers to match texture properties (rather than specific samples as in Experiments 1 and 2) of real and synthetic textures subject to changing vs. stable illumination. In all three experiments, we observe a significant cost of synthetic appearance, suggesting that the summary statistics included in the P-S model do not carry sufficient information to account for observers' abilities to match textures in an invariant way.

## Experiment 1

In our first task, we used the Portilla-Simoncelli model as a means of determining the extent to which a rich appearance code based on summary-statistics was sufficient to support observers' ability to match texture samples under illumination change.

### Method

#### Subjects

We recruited 13 participants (5 female) from the NDSU Introductory Psychology study pool. All participants reported normal or corrected-to-normal vision and received course credit for volunteering. Participants were between the ages of 18-25 years old and were naïve to the purpose of the experiment. In this experiment (and also Experiments 2 and 3) we obtained written informed consent from all participants and all three experiments were conducted in accordance with the ethical principles outlined in the Declaration of Helsinki.

#### Stimuli

**Figure 1:**
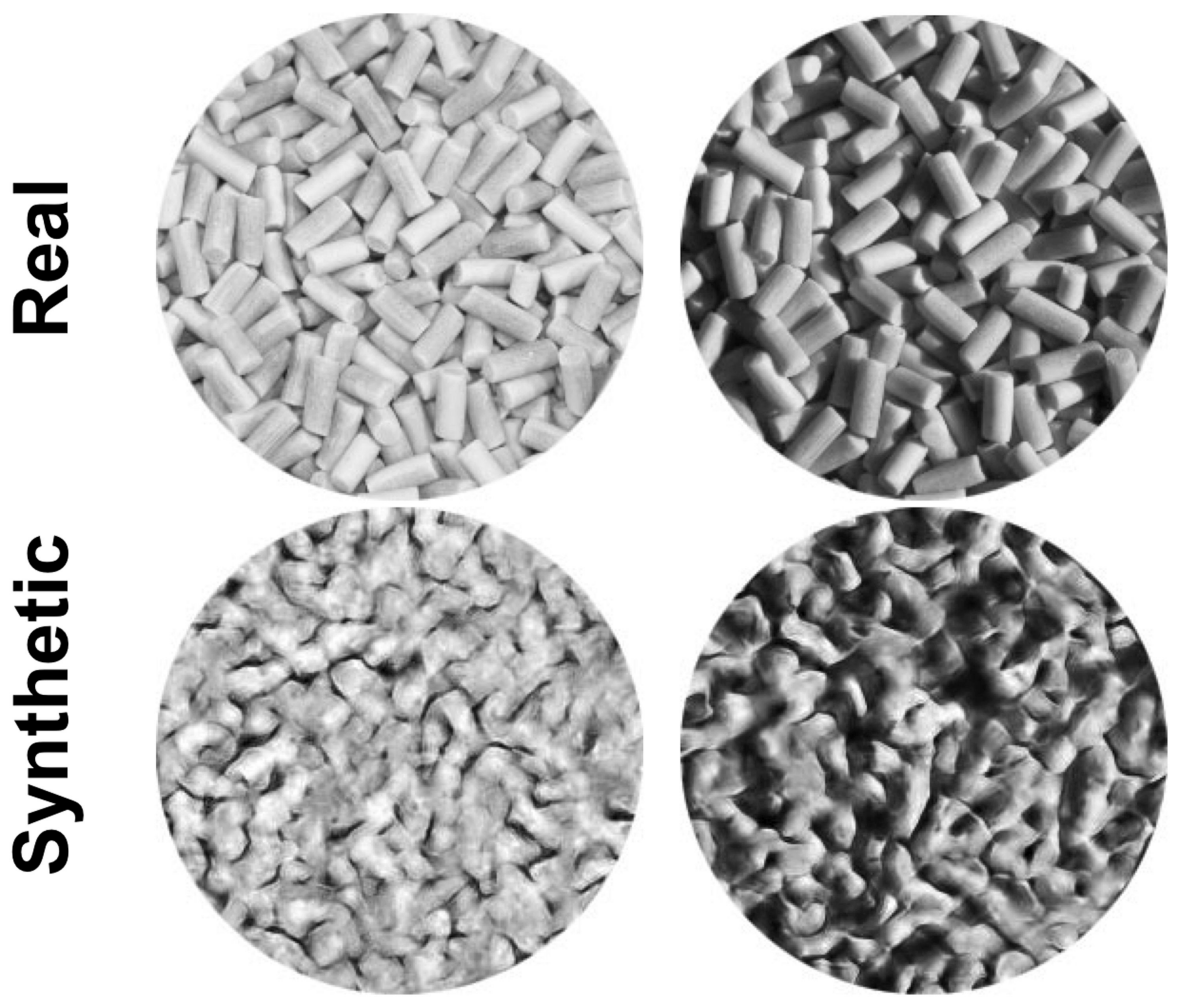
Examples of our texture stimuli, including both original and synthetic examples made from images with diffuse overhead lighting and side lighting.

Our stimulus set was comprised of 14 textures selected from the Amsterdam Library of Textures (Burghouts & Geusebroek, 2009), each photographed under diffuse overhead lighting and strong side lighting (Figure 1). We selected images based on the availability of visually-matched pairs in the database, such that each texture we selected had a counterpart in the stimulus set that was approximately matched for mean luminance, contrast, the spatial scale of texture elements, etc. The original texture images were 384 × 256 pixels in size and rendered in grayscale.

We created synthetic versions of each original texture by applying the Portilla-Simoncelli model (Portilla & Simoncelli, 2000). This model describes texture appearance primarily via correlations between wavelet coefficients that capture joint statistics across position, scale, and orientation (see Balas, Nakano, & Rosenholtz, 2009 for a more thorough description of the features in the model). We generated synthetic textures using 4 orientations, 4 spatial scales, a spatial neighborhood of 21 pixels and 50 iterations of the algorithm for matching statistics between the target texture and the synthetic image. We chose these particular parameter values since these led to high-quality syntheses for all of the natural textures we selected - increasing the fidelity of the representation by increasing the number of scales and orientations did not lead to obvious differences in texture quality, and we found that decreasing any of the aforementioned parameters tended to lead to noticeably poorer synthetic images for some of our original textures (but not all). As a result, we opted to use a common set of parameter values that yielded high-quality synthetic images. Our final synthetic images were matched to the size of the original parent images by cropping them after synthesis.

Finally, we cropped circular texture patches that were 256 pixels in diameter from the original and synthetic images for use in our texture matching tasks. We note that cropping these patches after performing texture synthesis means that patches taken from the same parent image are not guaranteed to have texture statistics that are perfectly matched. This is also true of real textures, however, since natural images are also not perfectly stationary. In general, the texture statistics from different patches of the same parent image are likely to be more similar than the statistics of two patches drawn from different parent images unless we are considering a texture with substantial inhomogeneity (in which case different patches may have very different statistics) or if we working with pairs of distinct textures that are highly similar. We attempted to choose our original set of textures such that neither of these were the case, and our full set of synthetic stimuli is available in the Supplemental Materials. The ALOT database is freely available at: http://aloi.science.uva.nl/public_alot/ and the original texture images used here can be obtained there (the synthetic images we have included are numbered to correspond with the numbering used in the ALOT database).

#### Procedure

Participants were asked to complete a match-to-sample task using the texture patches described above. On each trial, participants were instructed to observe the sample texture presented first, and identify which of two subsequently presented patches depicted the same texture as the sample. In the *illumination-change* condition, the matching test image and the sample image were illuminated differently. In the *no-change* condition, the matching test image and the sample image were identical. In both cases, the non-matching test image on each trial depicted a texture patch drawn from the texture that was selected to be an approximately visually-matched foil for the sample texture. Participants completed the *illumination-change* and *no-change* conditions in separate blocks, with block order counterbalanced across observers. Within a block, texture appearance (real or synthetic), the left/right position of the matching test texture, and the illumination of the sample texture (overhead or side illumination) were pseudo-randomized.

**Figure 2:**
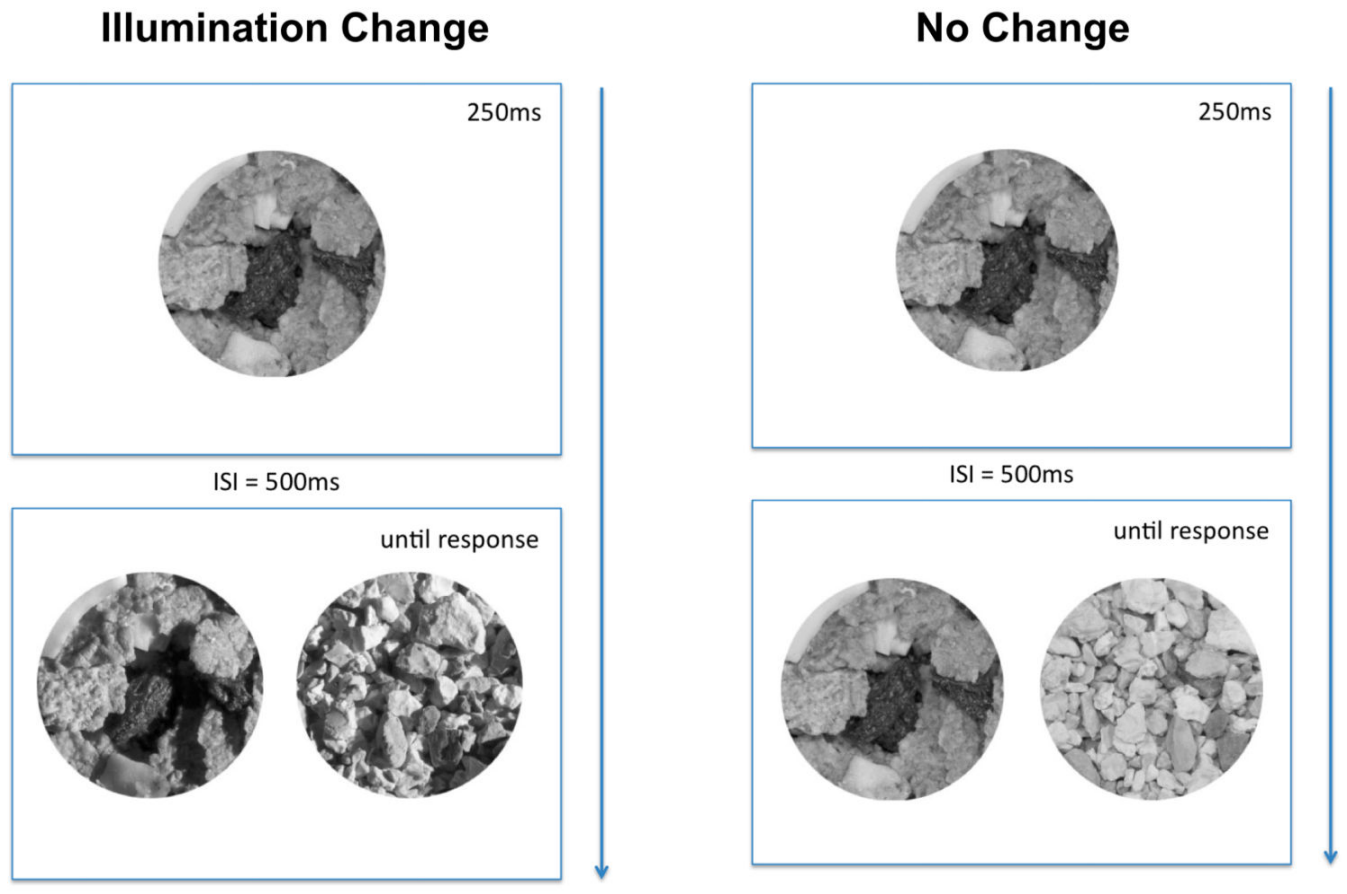
A schematic representation of a single trial of our match-to-sample task. Participants were asked to select the test image on each trial that was drawn from the same texture as the sample image.

Participants viewed the stimuli at a distance of 40cm on a 1024×768 LCD display. At this viewing distance each texture patch subtended approximately 3 degrees at this distance. On each trial, the sample texture was presented on a black background for 250ms, followed by an ISI of 500ms and the two test images presented on a black background until response (Figure 2). Participants indicated which test image matched the sample using the left and right shift keys. We recorded response accuracy and reaction time -participants were asked to respond as quickly as possible while still being accurate. Participants completed a total of 224 trials per block, for a grand total of 448 trials in the entire experimental session. Participants typically completed the entire task in approximately 30 minutes. All stimulus display and response collection routines were implemented in the MATLAB Psychophysics toolbox (Brainard, 1997; Pelli, 1997).

### Results and Discussion

For each participant we calculated the proportion of correct responses in both the *illumination-change* and *no-change* conditions for real and synthetic textures. We also calculated median reaction time for correct responses. We analyzed both measures using a 2×2 repeated-measures ANOVA with lighting condition (illumination change vs. no change) and texture appearance (real vs. synthetic) as within-subject factors.

#### Accuracy

**Figure 3:**
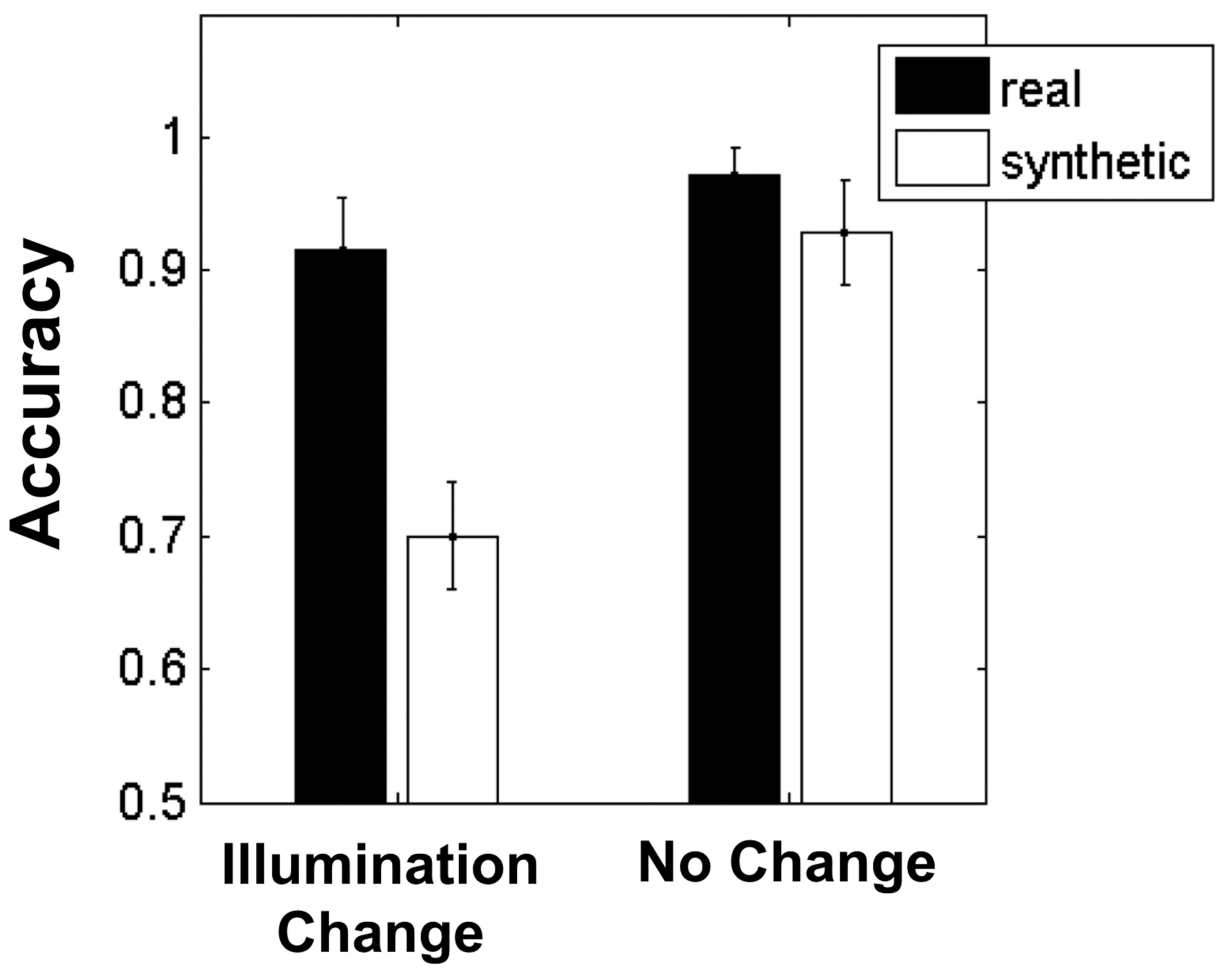
Average proportion correct across all participants as a function of task (illumination change vs. no change) and texture appearance (real vs. synthetic). Error bars represent +/- 1 s.e.m.

We observed significant main effects of lighting condition (F(1,12)=207.4, p<0.001, η^2^=0.94) and texture appearance (F(1,12)=326.0, p<0.001, η^2^=0.96) on participants' accuracy in our task. The former was the result of greater accuracy in the *no-change* condition (M=95.0, 95% CI = [93.2,96.8]) relative to the *illumination change* condition (M=80.8%, 95% CI = [78.7,82.9]). The latter was driven by greater accuracy for matching real textures (M=94.3%, 95% CI=[92.7,96.0]) than synthetic ones (M=81.4%, 95% CI=[79.5,83.4]). These main effects were also qualified by a significant interaction between lighting condition and texture appearance (F(1,12)=92.3, p<0.001, η^2^=0.89). The interaction was the result of a larger difference between real and synthetic texture accuracy in the *illumination change* condition (M_real_=91.5%, M_synthetic_=70.1%) than in the *no-change* condition (M_real_=97.2%, M_synthetic_=92.8%). Though this difference is smaller in the *no-change* condition, it is nonetheless still statistically significant (t(12)=4.48, p=0.001, two-tailed paired-samples t-test). The average accuracy per condition across all participants is displayed in Figure 3.

#### Reaction time

We also observed main effects of lighting condition (F(1,12)=15.6, p=0.002, η^2^=0.56) and texture appearance (F(1,12)=6.36, p=0.027, η^2^=0.35) on the median reaction time to correct responses. The main effect of lighting condition was the result of slower reaction times in the *illumination change* condition (M=0.74, 95% CI=[0.64,0.86]) than in the *no-change* condition (M=0.61, 95% CI=[0.55 0.68]). The main effect of texture appearance was the result of slower reaction times in response to synthetic textures (M=0.72, 95% CI=[0.62,0.83]) than to real textures (M=0.63, 95% CI=[0.56, 0.76]). We observed no interaction between these factors (F(1,12)=2.50, p=0.14, η^2^=0.17).

### Discussion

We conclude from this experiment that the P-S algorithm appears to be insufficient for to account for observers' ability to match texture samples given the relatively mild changes in appearance that we have considered here (illumination change). Specifically, the fact that we observed not only a significant cost of synthetic appearance when illumination invariance was required, but also a *larger* cost than when it was not suggests that this set of texture descriptors lacks information that is useful for matching textures across a lighting change. The no change condition we have used serves as a way to evaluate the quality of the synthetic images we used here, ensuring that poor performance with synthetic textures in the illumination-change condition is not simply the result of such poor texture synthesis that all of our textures appear highly similar to one another once they are synthesized. Were this the case, we would expect that synthetic textures should be hard to match in all cases due to high inter-texture similarity. Instead, we observed that the cost was especially high when illumination invariance was required, suggesting that our synthetic textures were of sufficiently high quality to support texture matching under some conditions. We point out, however, that since the no-change condition could theoretically be accomplished using image-based features (pixels), we cannot draw strong conclusions about texture processing on the basis of this control. Presently, we simply use it as an indicator of what our observers can do with these synthetic textures under optimal conditions for matching - an “upper bound” on performance with these images.

One potential weakness of this task is that we did not control for low-level properties of the texture images like the mean luminance of the sample and test images or the contrast of these image. One possible account of the large cost for synthetic textures in the *illumination change* condition is that these properties contribute substantially to observers' performance with real textures and are somehow less available in synthetic textures (or less useful, since the P-S algorithm only matches moments of the intensity histogram rather than the entire function and may thus leave out some information). Indeed, Groen et al. (2012) recently demonstrated that variation in low-level contrast was predictive of natural texture invariance, suggesting that illumination invariance may be supported in part by these properties of real-world images. Thus, to examine the role of these basic features in the *illumination change* condition, we implemented a second task with mean luminance, contrast, and the power spectrum matched across our entire stimulus set.

## Experiment 2

In our second task, we re-ran our *illumination-change* condition after applying luminance, contrast and spatial frequency matching to our entire stimulus set. To the extent that low-level differences in mean luminance and contrast following texture synthesis may have contributed to the disproportionate performance cost we observed in this task in Experiment 1, matching these properties across our stimulus set may reduce the impact of texture synthesis on observers' ability to match texture samples when illumination changes.

### 

#### Subjects

We recruited an additional group of 13 participants (4 female) for this task, none of whom had taken part in Experiment 1. All of these participants reported normal or corrected-to-normal vision and were naïve to the purpose of the experiment. We obtained written informed consent from all participants prior to their participation in the task.

#### Stimuli

**Figure 4:**
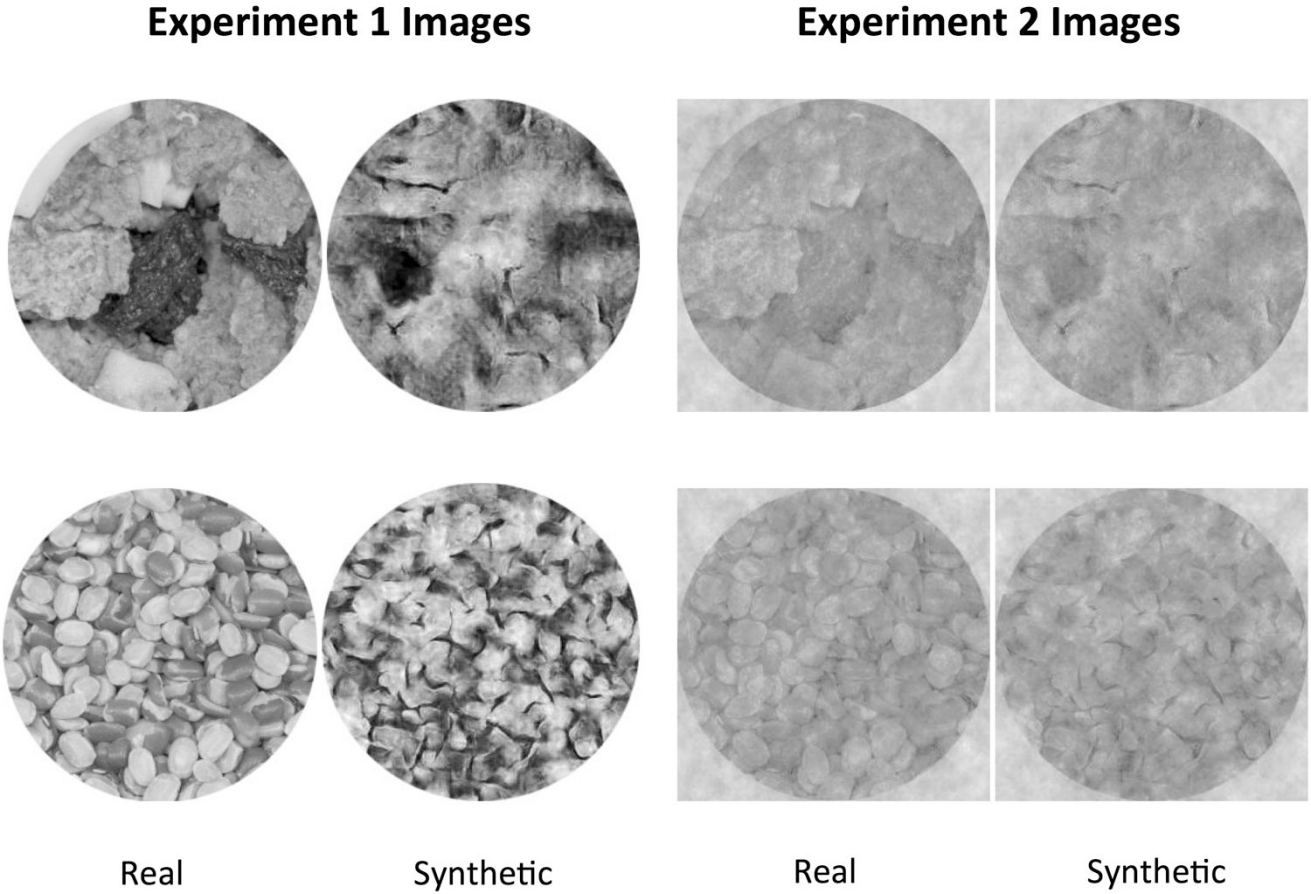
Examples of the power-spectrum matched textures used in Experiment 2 compared to the original image patches from Experiment 1.

The same stimulus set described above in Experiment 1 was used here. However, to ensure that mean luminance and contrast were matched across our images, we used the SHINE toolbox (Willenbockel et al., 2010) to enforce a common mean luminance and a common average power spectrum across our entire stimulus set. This was applied to the patches cropped from the original textures to ensure that each texture patch closely matched low-level properties. In Figure 4 we display examples of real and synthetic images from Experiment 1 before and after this transformation was applied.

#### Procedure

All display parameters and experimental procedures were identical to those described above in Experiment 1, with the only caveat being that participants in this task were not asked to complete a *no-change* block.

### Results

We computed each participant's response accuracy and median reaction time for correct responses for both real and synthetic trials. In each case, we compared performance for real and synthetic textures using a two-tailed paired-samples t-test. We observed a significant difference in accuracy for real and synthetic textures (t(12)=15.1, p<0.001), such that performance with real textures (M=82.8%, 95% CI=[78.6, 86.5]) was more accurate than performance with synthetic textures (M=64.6%, 95% CI=[61.3, 67.8]). Similarly, median reaction times for correct responses were also significantly different for real and synthetic textures (t(12)=3.97, p=0.002) such that correct responses to real textures (M=0.68, 95% CI=[0.63,0.74]) were faster than correct response to synthetic textures (M=0.81, 95% CI=[0.71,0.91]). As in Experiment 1, the observation of poorer accuracy and slower RTs for synthetic textures suggest that our effects are not simply the result of a speed-accuracy trade-off but reflect less efficient processing of synthetic textures. We also note that the approximate cost of using synthetic textures in this implementation of the *illumination change* condition (∼18 percentage points) is very close to the cost observed when we did not closely control mean luminance and contrast (∼21 percentage points), which could be interpreted to mean that our initial results were not simply the result of an improved ability to use these properties in natural images to achieve illumination invariance. However, we also point out that the absolute level of performance in this task is lower than observed in Experiment 1, meaning that the absolute difference between conditions should perhaps be considered along side something like the proportional difference.

### Discussion

Considered as a whole, the results from Experiments 1 and 2 suggest that invariance to illumination change when matching texture samples is not supported by the summary statistics used in the Portilla-Simoncelli model and that observers' relative success with natural textures does not appear to be driven by very basic low-level features. Were this the case, images matched for these properties by the SHINE algorithm should both be very difficult to match in general and also should have led to a different profile of results. Instead, we found in both experiments that texture synthesis incurred a significant (and large) performance cost when observers were required to achieve illumination invariance, but incurred a smaller cost when texture sample matching could be achieved without any need to cope with lighting changes.

Again, we do note one limitation of the design used in both experiments that weakens our ability to draw strong conclusions about texture processing *per se* based on these data. In both Experiment 1 and Experiment 2, the patches of real textures presented to observers as sample and test stimuli were taken from the same location in the parent texture. This means that though illumination may change across sample and test presentation, object-like structures are presented in the same location. This kind of matching is not possible in the synthetic appearance conditions, since syntheses of the same texture under different lighting conditions is not at al constrained such that proto-objects or objects are generated in the same positions. Thus, the difference between real and synthetic appearance in the illumination change condition could conceivably have little to do with texture processing proper and instead be the result of object processing applied to the object parts and surfaces that are preserved across illumination changes for real textures. Such processes would be far less useful in the synthetic appearance condition, meaning that the differences we have reported may reflect the efficacy of object processing rather than properties of texture processing. Similarly, the use of identical texture patches in the no-change condition makes it possible for participants to apply both position-dependent processes using raw pixel intensities and object-like processes in the real texture condition. Again, the effects of texture appearance could be driven by the successful application of a wide range of high-level processes in the real appearance condition. This possibility is to some extent even more likely given that we permitted observers to freely view the stimuli, rather than presenting them in the visual periphery (Balas, 2006). While peripheral presentation does not ensure that texture representations are all that are available to the observer, the ability to foveate texture patches in the current study makes a wide range of candidate mechanisms available to our observers. Thus, while it seems reasonable to conclude that texture processing may be the dominant mode when observer are viewing our synthetic textures, we concede that many processes may contribute when real textures were presented. We suggest that the results from Experiment 1 and 2 still tell us something about texture processing, since the effects of texture synthesis reflect the constraints imposed by a texture code, but we also acknowledge that it is difficult to interpret the data from our real textures given that participants could usefully apply many strategies and representations to these stimuli and meet with success.

To help constrain the processes observers can usefully apply to this task more closely, in Experiment 3 we minimized the potential for object-like processing and position-dependent processing to contribute to matching performance by drawing sample and test patches in all conditions from different locations in the parent image. This manipulation makes it substantially less useful for participants to use object parts as a means of matching textures across illumination changes and rules out strongly position-dependent representations as well. To the extent that we are able to replicate the results of our prior tasks using this method choosing image patches, we may draw stronger conclusions regarding the properties of texture processing in particular.

## Experiment 3

In our final experiment, we attempted to replicate the results of Experiment 1 using patches of sample and test textures that did not come from the same location in the original parent image. This manipulation was designed to minimize the contribution of position-dependent strategies and object-like processing that could potentially have supported performance in Experiment 1 by using non-overlapping patches from the parent texture images. As such, this final experiment makes it possible for us to comment on observers' ability to match texture properties, rather than samples, across an illumination change.

### 

#### Subjects

We recruited an additional 12 observers (8 female) to participate in this task. None of these participants had taken part in either Experiment 1 or Experiment 2. All participants reported either normal or corrected-to-normal vision and also reported that they were free of neurological or visual impairments. As in Experiments 1 and 2, we obtained written informed consent from all participants prior to their participation in the task.

#### Stimuli

We used the same stimuli described in Experiment 1, with the exception of the procedural differences in selecting sample and test patches described below.

#### Procedure

All testing procedures were identical to those described in Experiment 1, save for the selection of sample and test images on each trial. To help ensure that participants were completing the task using texture-like processes rather than either position-dependent mechanisms or object-like processes, we chose the sample image and its corresponding test image such that they did not overlap. This required the use of larger parent images than those used for Experiment 1 (these were 768 × 512), from which samples 256×256 in size were sampled such that patches did not overlap. In this way participants were not able to identify specific local features or object parts that would be present in both the sample image and its matching test image. To further minimize the extent to which position-dependent mechanisms might be used to support performance in this task, we also rotated all test images 45 degrees clockwise relative to the sample, further reducing the possibility that observers would be able to use pixel-based strategies to support performance. Aside from these manipulations, display parameters and task design were unchanged.

### Results

As in Experiment 1, we computed the average accuracy per stimulus condition for all participants as well as their average response time for correct responses. In each case, we submitted these values to a 2×2 repeated-measures ANOVA with texture category (real vs. synthetic) and illumination condition (illumination change vs. no change) as within-subject factors.

#### Accuracy

This analysis revealed main effects of texture category (F(1,11)=115.7, p<0.001, partial eta-squared = 0.91) and of illumination condition (F(1,11)=81.9, p<0.001, partial eta-squared =0.88). We also observed a significant interaction between these factors (F(1,11)=53.5, p<0.001, partial eta-squared = 0.83). The main effect of texture category was driven by greater accuracy for real textures (M=0.94, s.e.m.=0.01) than synthetic textures (M=0.76, s.e.m.=0.02), while the main effect of illumination condition was driven by greater accuracy in the no-change condition (M=0.93, s.e.m.=0.01) than in the illumination condition (M=0.78, s.e.m.=0.02).

To examine the interaction between these factors more closely, we first carried out post-hoc comparisons between the results obtained from real and synthetic textures in each illumination condition. In both cases, we observed significant differences favoring real textures (Illumination change condition: t(11)=9.56, p<0.001, two-tailed, paired-samples t-test); No change condition: t(11)=7.06, p<0.001, two-tailed paired-samples t-test). Next, we compared participants' accuracy in the illumination-change vs. no-change condition for both real and synthetic textures. This analysis revealed significantly poorer performance in the illumination change condition when synthetic textures were used (t(11)=-9.16, p<0.001) but only a marginal difference (after applying a Bonferroni correction for multiple tests) when real textures were used (t(11)=-2.63, p=0.023). We suggest that the interaction we observed is thus the result of substantially poor performance in the synthetic condition when illumination changes. To facilitate comparison across all three experiments, the accuracy scores in each condition, along with accompanying 95% confidence intervals are displayed in Table 1, along with the scores we observed in Experiments 1 and 2.

#### Response Times

Our analysis of response times to correct responses yielded results very similar to those obtained from our accuracy data. We observed main effects of both texture category (F(1,11)=7.99, p=0.016, partial eta-squared = 0.42) and illumination condition (F(1,11)=33.0, p<0.001, partial eta-squared = 0.75) and an interaction between these factors (F(1,11)=8.57, p=0.014, partial eta-squared = 0.44). The main effect of texture type was driven by slower responses to synthetic textures (M=0.64, s.e.m.=0.04) relative to real textures (M=0.55, s.e.m.=0.024) and the main effect of illumination condition was driven by slower responses in the illumination change condition (M=0.67, s.e.m.=0.04) than in the no-change condition (M=0.52, s.e.m.=0.02).

As above, we used post-hoc comparisons to examine the nature of the interaction we observed in the response time data. We compared response times in the real and synthetic texture conditions for both the illumination-change task and the no-change task. In the former case, we observed a significant difference between real and synthetic textures (t(11)=-3.02, p=0.012, two-tailed paired-samples t-test) but no such difference in the latter comparison (t(11)=-.49, p=0.64). This suggests that in this case, synthetic appearance leads to a performance cost in the illumination change condition, but does not affect performance when there is no need for illumination-invariant processing.

## General Discussion

Our results demonstrate that the summary statistics implemented in the Portilla-Simoncelli model are not sufficient to capture observers' ability to match texture samples (Experiments 1 and 2) or texture properties across different samples (Experiment 3) in an illumination-invariant fashion. Were this the case, synthesizing our original images should have preserved the necessary similarity relationships between textures lit from overhead and from the side such that identifying the matching test images remained straightforward. Instead, we observed both a significant cost for synthesizing textures when illumination changes needed to be considered and also a cost for synthesizing textures when pixel-level comparisons were enough to yield the right answer (our *no-change* condition). Both of these results were obtained under conditions where position-dependent processes and object-like processes could theoretically be used to achieve accurate performance (Experiments 1 and 2) and also under conditions where such mechanisms were not as useful (Experiment 3) and texture processing is more likely to be the primary means of completing the task. Our latter result (poorer performance with synthetic textures when there is no lighting change) is consistent with data reported in Balas (2012) describing a similar impact of synthetic appearance on observers' ability to detect an oddball texture patch among an array of distractors. Impressive sensitivity to natural texture structure is evident in several other recent studies: Gerhard, Wichmann, & Bethge (2013) demonstrated via a generative patch-based model of texture appearance that observers were able to quickly learn to discriminate model patches from natural patches, for example, and that current models of image structure do not adequately capture the high-order statistics observers appear to have access to. Similarly, Emrith et al. (2010) quantified observers' sensitivity to high-order statistics using a phase-scrambling technique, identifying a critical amount of phase randomization that observes were able to detect. The current results from the *no-change* condition thus add to a growing literature demonstrating how high-order statistics in natural images contribute to texture perception in multiple settings. Whether such effects result from some aspect of texture processing itself or some more general property of mid-level or high-level vision remains an open question, but the current study offers some useful constraints on what can and cannot be achieved with a rich texture code for appearance.

Our data from this relatively simple task indicate that the Portilla-Simoncelli model does not offer an adequate description of the features that support invariant texture perception, and may in general not constrain synthetic textures to have sufficiently naturalistic structure for efficient visual processing (though see Freeman et al., 2013 for fMRI data suggesting that P-S textures drive V2 responses). While the current study was not designed explicitly to test material perception, shape-from-texture, or other diverse functions texture perception subserves, it is nonetheless instructive to discuss these effects insofar as they also indicate some important cases in which it seems a purely summary-statistic code likely cannot capture observers' judgments about textures. Similar to the results by Ho, Landy & Maloney described in the introduction, a further study by the same group (Ho, Landy, & Maloney, 2008) demonstrated confusability between perceived glossiness and surface relief, suggesting some further complexity between the measurements used to characterize glossiness and the depth information in the texture. As Fleming (2014) describes, further examination of this phenomenon by Marlow, Kim & Anderson (2012) revealed that properties of specular highlights including their size and distinctiveness appeared to account for the effect. This can be interpreted as evidence that besides a feed-forward summary-statistic representation of texture appearance, segmentation processes that allow observers to assess features like specular highlights effectively may play a critical role in a range of texture tasks. These various studies suggest an important role for additional computational mechanisms that contribute to texture perception by integrating information about local shape relationships, depth information, and other properties of the texture under consideration that can be derived from specific critical features following some process of perceptual organization. Arguably, one could imagine that a sufficiently rich set of summary statistics may be enough to describe the necessary properties that are relevant in these case, but presently, we suggest that the current study offers an important demonstration that a popular model of texture appearance is likely not up to this task.

One obvious limitation of the current study is our reliance on a specific model of texture appearance (the P-S algorithm). An easy criticism of these results is that the Portilla-Simoncelli algorithm is only one example of a large class of possible appearance models for texture, and other models may yield different results. This is a valid point and we do not disagree with the main thrust of this potential criticism – indeed, we suggest that continued investigation with both subsets of the P-S model (see Balas, 2006 for results using this technique) and other parametric texture synthesis models that relate easily to the human visual system (e.g. Heeger & Bergen, 1995) is an important means of understanding the scope of summary statistic representations for texture and material processing. The P-S algorithm has garnered substantial attention in recent years as a candidate model of texture processing (Balas, 2006; 2012), peripheral vision (Balas, Nakano & Rosenholtz, 2009; Rosenholtz et al., 2011), and V2 functionality (Freeman & Simoncelli, 2011; Freeman et al., 2013), making it a useful target model for the current study. However, examining other models may yield important insights into the capabilities and limitations of summary statistics as a tool for explaining human texture perception. A close consideration of a wider range of parameter values in the P-S model and/or texture representations that include either different visual features (explicit inclusion of center-surround features, or various forms of corner detectors, for example) or higher-order joint statistics of the same basic measurements that are the basis of the Portilla-Simoncelli algorithm may be an important avenue for understanding what image descriptors support robust performance in natural images that are subject to realistic variation in appearance. Similarly, the consideration of a wider range of ecologically valid transformations of texture appearance, such as viewpoint change or scale change, would also yield important information regarding how well specific models of texture appearance carry information sufficient for matching texture samples or properties in an invariant way. Finally, we also note (as we discussed in the introduction) that the current study does not apply synthetic textures in an attempt to approximate peripheral vision or to compare performance with synthetic images to peripherally-viewed textures. This is a potentially important avenue for further inquiry however, and whether or not synthetic approximations of texture appearance are adequate for explaining observer's abilities in the visual periphery is an open and important question.

## Supplementary Material

suppl.

## Figures and Tables

**Table 1:** Average accuracy per condition in all three experiments, with 95% confidence intervals.

	Real Textures	Synthetic Textures

Illumination Change – Exp 3	M=0.92; 95% CI=[0.89-0.95]	M=0.63; 95% CI=[0.56-0.71]
No Change – Exp 3	M=0.96; 95% CI=[0.94-0.98]	M=0.89; 95% CI=[0.87-0.92]
Illumination Change – Exp 2	M=0.83; 95% CI=[0.79-0.87]	M=0.65; 95% CI=[0.62-0.68]
Illumination Change – Exp 1	M=0.92; 95% CI=[0.89-0.94]	M=0.70; 95% CI=[0.68-0.72]
No Change – Exp 1	M=0.97; 95% CI=[0.96-0.98]	M=0.93; 95% CI=[0.90-0.96]

## Research Highlights

- Texture synthesis impairs texture sample matching across illumination changes.
- Synthesis also impairs illumination-invariant matching of texture properties.
- Statistical properties of real images are critical for invariant recognition.
