# Supplementary figures and images for: Invariant texture perception is harder with synthetic textures: Implications for models of texture processing (part 2 of 2)
Source: Vision Res. Author manuscript; Available in PMC 2016 Oct 1. (PMC4529380; doi:10.1016/j.visres.2015.01.022)

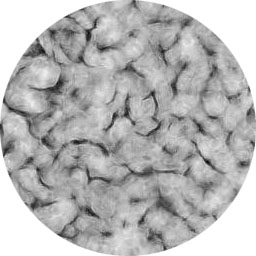

Supplement: suppl. [file NIHMS662495-supplement-suppl_.zip › BalasConlin_SyntheticTextures/76s.1_left.jpg]

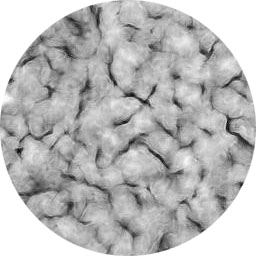

Supplement: suppl. [file NIHMS662495-supplement-suppl_.zip › BalasConlin_SyntheticTextures/76s.1_right.jpg]

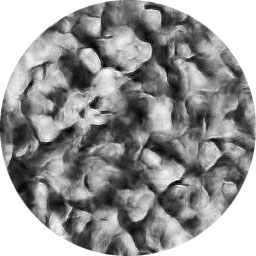

Supplement: suppl. [file NIHMS662495-supplement-suppl_.zip › BalasConlin_SyntheticTextures/76s.2_left.jpg]

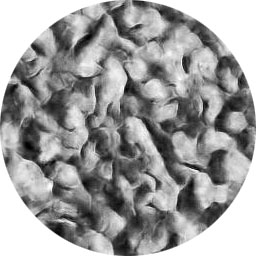

Supplement: suppl. [file NIHMS662495-supplement-suppl_.zip › BalasConlin_SyntheticTextures/76s.2_right.jpg]

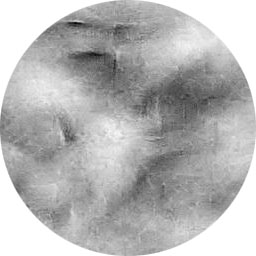

Supplement: suppl. [file NIHMS662495-supplement-suppl_.zip › BalasConlin_SyntheticTextures/98s.1_left.jpg]

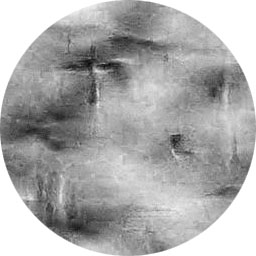

Supplement: suppl. [file NIHMS662495-supplement-suppl_.zip › BalasConlin_SyntheticTextures/98s.1_right.jpg]

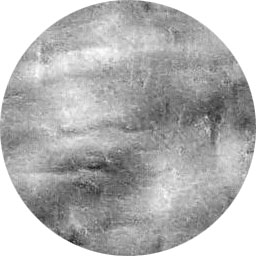

Supplement: suppl. [file NIHMS662495-supplement-suppl_.zip › BalasConlin_SyntheticTextures/98s.2_left.jpg]

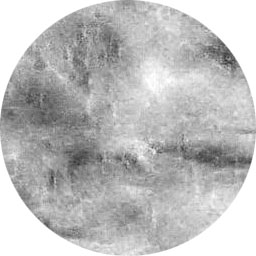

Supplement: suppl. [file NIHMS662495-supplement-suppl_.zip › BalasConlin_SyntheticTextures/98s.2_right.jpg]
