# Supplementary figures and images for: Invariant texture perception is harder with synthetic textures: Implications for models of texture processing (part 1 of 2)
Source: Vision Res. Author manuscript; Available in PMC 2016 Oct 1. (PMC4529380; doi:10.1016/j.visres.2015.01.022)

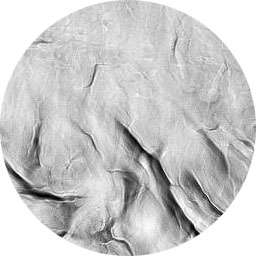

Supplement: suppl. [file NIHMS662495-supplement-suppl_.zip › BalasConlin_SyntheticTextures/101s.1_left.jpg]

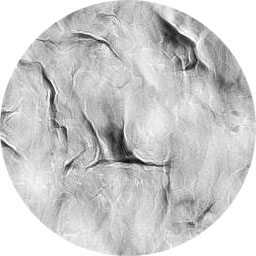

Supplement: suppl. [file NIHMS662495-supplement-suppl_.zip › BalasConlin_SyntheticTextures/101s.1_right.jpg]

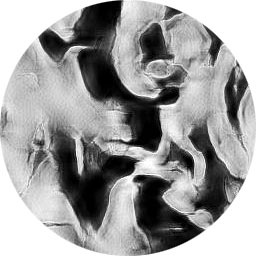

Supplement: suppl. [file NIHMS662495-supplement-suppl_.zip › BalasConlin_SyntheticTextures/101s.2_left.jpg]

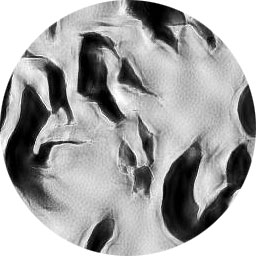

Supplement: suppl. [file NIHMS662495-supplement-suppl_.zip › BalasConlin_SyntheticTextures/101s.2_right.jpg]

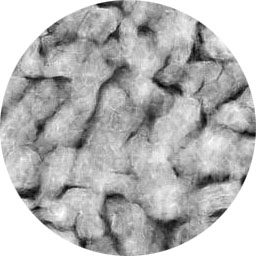

Supplement: suppl. [file NIHMS662495-supplement-suppl_.zip › BalasConlin_SyntheticTextures/112s.1_left.jpg]

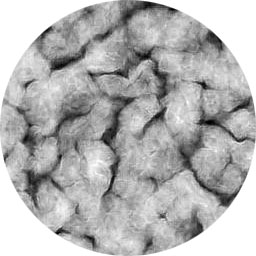

Supplement: suppl. [file NIHMS662495-supplement-suppl_.zip › BalasConlin_SyntheticTextures/112s.1_right.jpg]

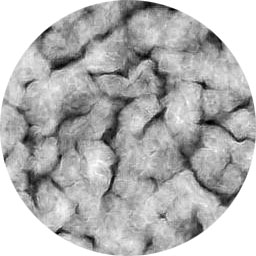

Supplement: suppl. [file NIHMS662495-supplement-suppl_.zip › BalasConlin_SyntheticTextures/112s.2_left.jpg]

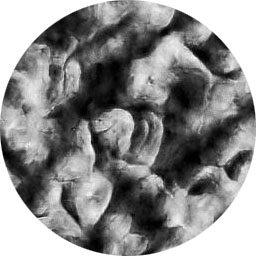

Supplement: suppl. [file NIHMS662495-supplement-suppl_.zip › BalasConlin_SyntheticTextures/112s.2_right.jpg]

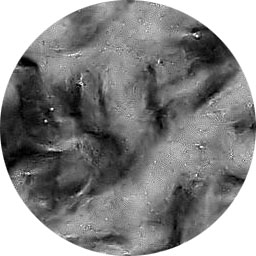

Supplement: suppl. [file NIHMS662495-supplement-suppl_.zip › BalasConlin_SyntheticTextures/119s.1_left.jpg]

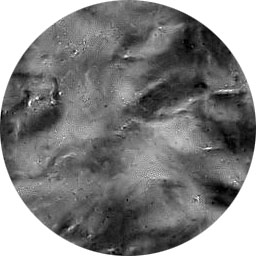

Supplement: suppl. [file NIHMS662495-supplement-suppl_.zip › BalasConlin_SyntheticTextures/119s.1_right.jpg]

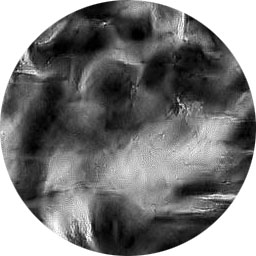

Supplement: suppl. [file NIHMS662495-supplement-suppl_.zip › BalasConlin_SyntheticTextures/119s.2_left.jpg]

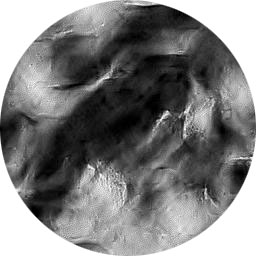

Supplement: suppl. [file NIHMS662495-supplement-suppl_.zip › BalasConlin_SyntheticTextures/119s.2_right.jpg]

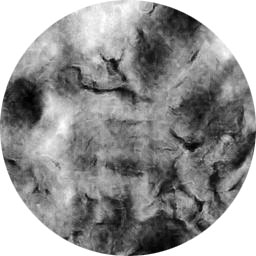

Supplement: suppl. [file NIHMS662495-supplement-suppl_.zip › BalasConlin_SyntheticTextures/11s.1_left.jpg]

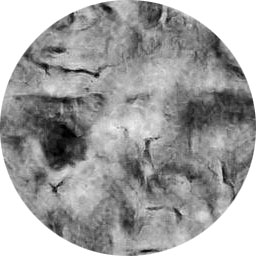

Supplement: suppl. [file NIHMS662495-supplement-suppl_.zip › BalasConlin_SyntheticTextures/11s.1_right.jpg]

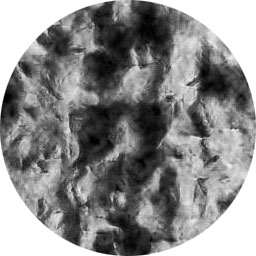

Supplement: suppl. [file NIHMS662495-supplement-suppl_.zip › BalasConlin_SyntheticTextures/11s.2_left.jpg]

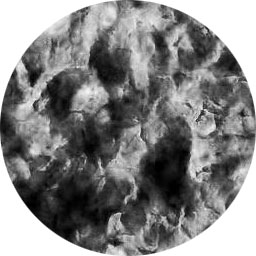

Supplement: suppl. [file NIHMS662495-supplement-suppl_.zip › BalasConlin_SyntheticTextures/11s.2_right.jpg]

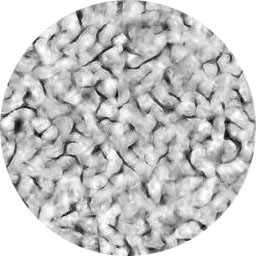

Supplement: suppl. [file NIHMS662495-supplement-suppl_.zip › BalasConlin_SyntheticTextures/125s.1_left.jpg]

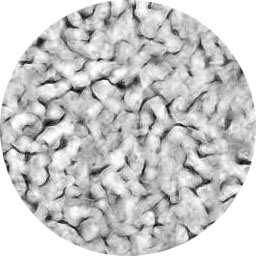

Supplement: suppl. [file NIHMS662495-supplement-suppl_.zip › BalasConlin_SyntheticTextures/125s.1_right.jpg]

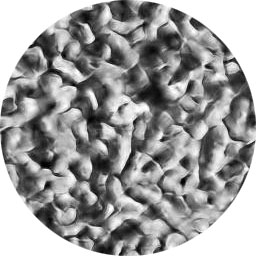

Supplement: suppl. [file NIHMS662495-supplement-suppl_.zip › BalasConlin_SyntheticTextures/125s.2_left.jpg]

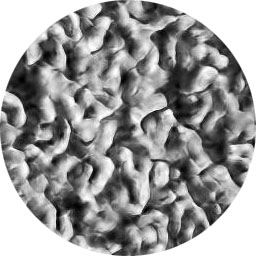

Supplement: suppl. [file NIHMS662495-supplement-suppl_.zip › BalasConlin_SyntheticTextures/125s.2_right.jpg]

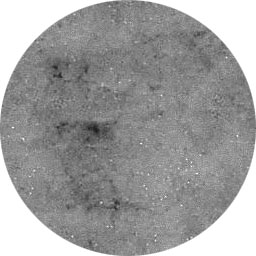

Supplement: suppl. [file NIHMS662495-supplement-suppl_.zip › BalasConlin_SyntheticTextures/12s.1_left.jpg]

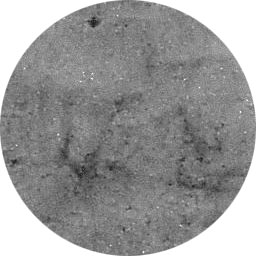

Supplement: suppl. [file NIHMS662495-supplement-suppl_.zip › BalasConlin_SyntheticTextures/12s.1_right.jpg]

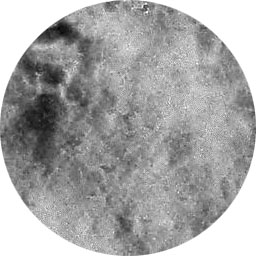

Supplement: suppl. [file NIHMS662495-supplement-suppl_.zip › BalasConlin_SyntheticTextures/12s.2_left.jpg]

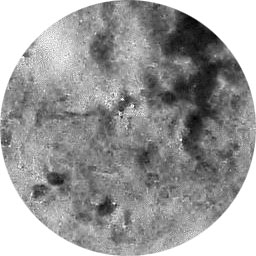

Supplement: suppl. [file NIHMS662495-supplement-suppl_.zip › BalasConlin_SyntheticTextures/12s.2_right.jpg]

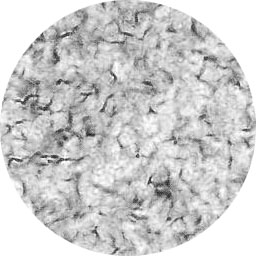

Supplement: suppl. [file NIHMS662495-supplement-suppl_.zip › BalasConlin_SyntheticTextures/139s.1_left.jpg]

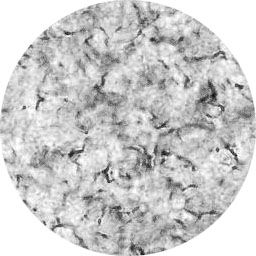

Supplement: suppl. [file NIHMS662495-supplement-suppl_.zip › BalasConlin_SyntheticTextures/139s.1_right.jpg]

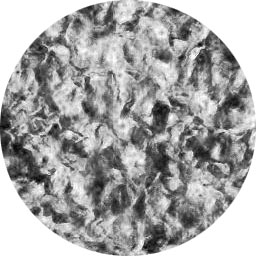

Supplement: suppl. [file NIHMS662495-supplement-suppl_.zip › BalasConlin_SyntheticTextures/139s.2_left.jpg]

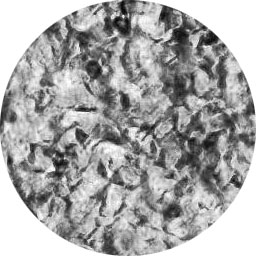

Supplement: suppl. [file NIHMS662495-supplement-suppl_.zip › BalasConlin_SyntheticTextures/139s.2_right.jpg]

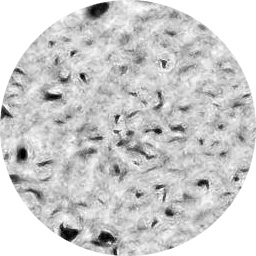

Supplement: suppl. [file NIHMS662495-supplement-suppl_.zip › BalasConlin_SyntheticTextures/140s.1_left.jpg]

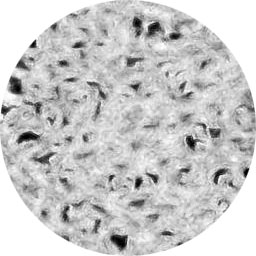

Supplement: suppl. [file NIHMS662495-supplement-suppl_.zip › BalasConlin_SyntheticTextures/140s.1_right.jpg]

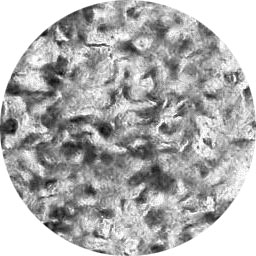

Supplement: suppl. [file NIHMS662495-supplement-suppl_.zip › BalasConlin_SyntheticTextures/140s.2_left.jpg]

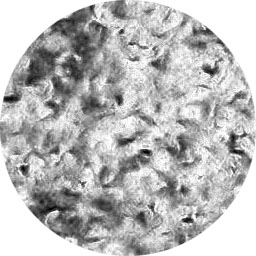

Supplement: suppl. [file NIHMS662495-supplement-suppl_.zip › BalasConlin_SyntheticTextures/140s.2_right.jpg]

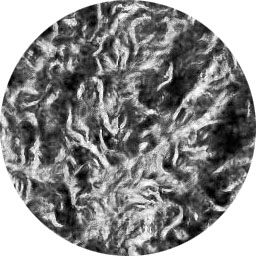

Supplement: suppl. [file NIHMS662495-supplement-suppl_.zip › BalasConlin_SyntheticTextures/145s.1_left.jpg]

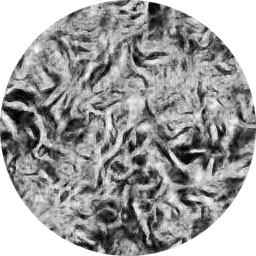

Supplement: suppl. [file NIHMS662495-supplement-suppl_.zip › BalasConlin_SyntheticTextures/145s.1_right.jpg]

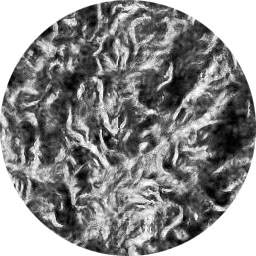

Supplement: suppl. [file NIHMS662495-supplement-suppl_.zip › BalasConlin_SyntheticTextures/145s.2_left.jpg]

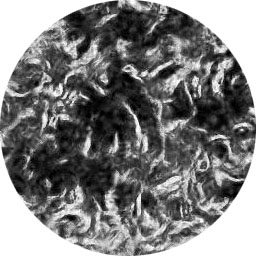

Supplement: suppl. [file NIHMS662495-supplement-suppl_.zip › BalasConlin_SyntheticTextures/145s.2_right.jpg]

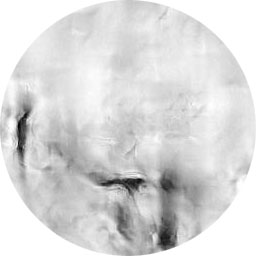

Supplement: suppl. [file NIHMS662495-supplement-suppl_.zip › BalasConlin_SyntheticTextures/167s.1_left.jpg]

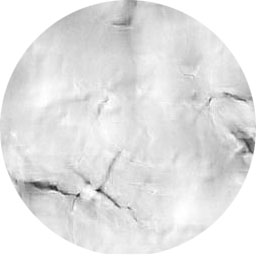

Supplement: suppl. [file NIHMS662495-supplement-suppl_.zip › BalasConlin_SyntheticTextures/167s.1_right.jpg]

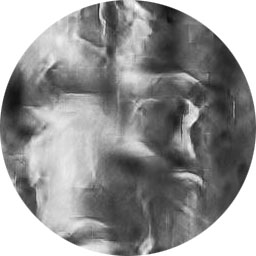

Supplement: suppl. [file NIHMS662495-supplement-suppl_.zip › BalasConlin_SyntheticTextures/167s.2_left.jpg]

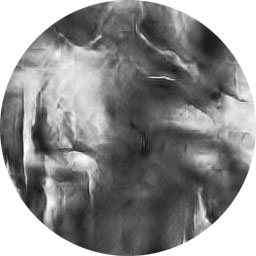

Supplement: suppl. [file NIHMS662495-supplement-suppl_.zip › BalasConlin_SyntheticTextures/167s.2_right.jpg]

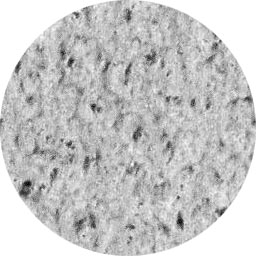

Supplement: suppl. [file NIHMS662495-supplement-suppl_.zip › BalasConlin_SyntheticTextures/176s.1_left.jpg]

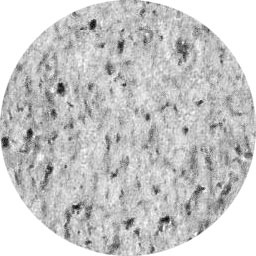

Supplement: suppl. [file NIHMS662495-supplement-suppl_.zip › BalasConlin_SyntheticTextures/176s.1_right.jpg]

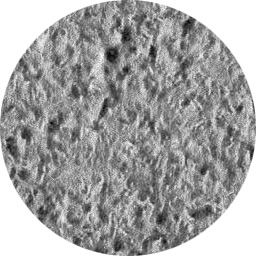

Supplement: suppl. [file NIHMS662495-supplement-suppl_.zip › BalasConlin_SyntheticTextures/176s.2_left.jpg]

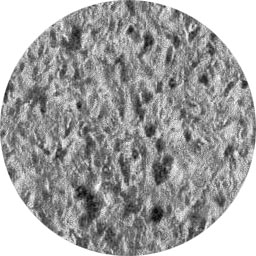

Supplement: suppl. [file NIHMS662495-supplement-suppl_.zip › BalasConlin_SyntheticTextures/176s.2_right.jpg]

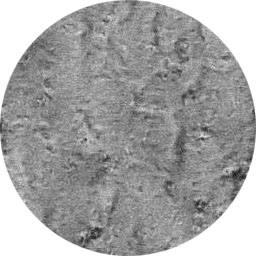

Supplement: suppl. [file NIHMS662495-supplement-suppl_.zip › BalasConlin_SyntheticTextures/178s.1_left.jpg]

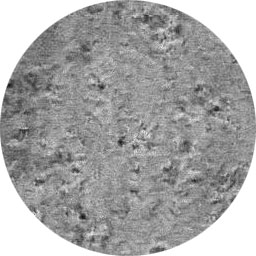

Supplement: suppl. [file NIHMS662495-supplement-suppl_.zip › BalasConlin_SyntheticTextures/178s.1_right.jpg]

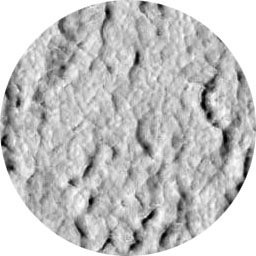

Supplement: suppl. [file NIHMS662495-supplement-suppl_.zip › BalasConlin_SyntheticTextures/178s.2_left.jpg]

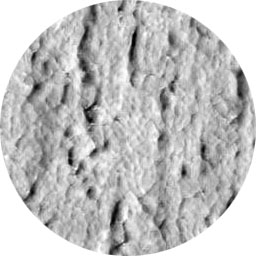

Supplement: suppl. [file NIHMS662495-supplement-suppl_.zip › BalasConlin_SyntheticTextures/178s.2_right.jpg]

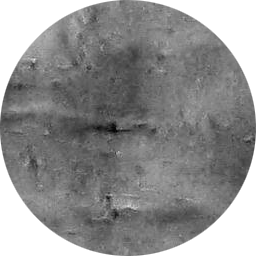

Supplement: suppl. [file NIHMS662495-supplement-suppl_.zip › BalasConlin_SyntheticTextures/186s.1_left.jpg]

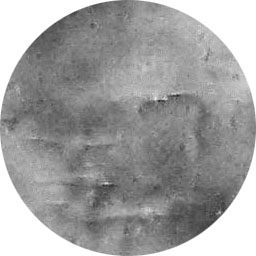

Supplement: suppl. [file NIHMS662495-supplement-suppl_.zip › BalasConlin_SyntheticTextures/186s.1_right.jpg]

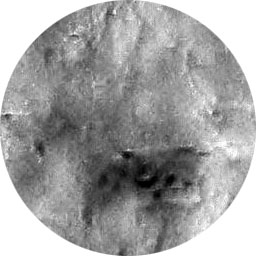

Supplement: suppl. [file NIHMS662495-supplement-suppl_.zip › BalasConlin_SyntheticTextures/186s.2_left.jpg]

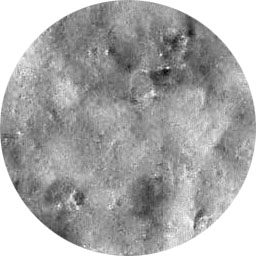

Supplement: suppl. [file NIHMS662495-supplement-suppl_.zip › BalasConlin_SyntheticTextures/186s.2_right.jpg]

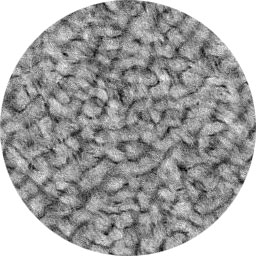

Supplement: suppl. [file NIHMS662495-supplement-suppl_.zip › BalasConlin_SyntheticTextures/205s.1_left.jpg]

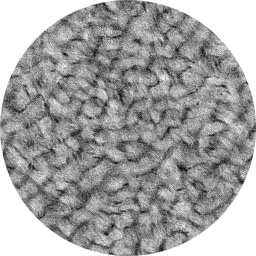

Supplement: suppl. [file NIHMS662495-supplement-suppl_.zip › BalasConlin_SyntheticTextures/205s.1_right.jpg]

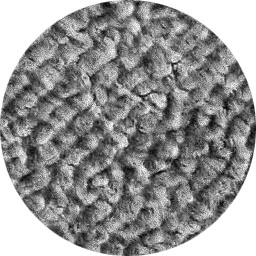

Supplement: suppl. [file NIHMS662495-supplement-suppl_.zip › BalasConlin_SyntheticTextures/205s.2_left.jpg]

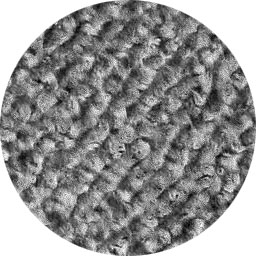

Supplement: suppl. [file NIHMS662495-supplement-suppl_.zip › BalasConlin_SyntheticTextures/205s.2_right.jpg]

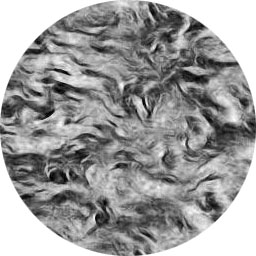

Supplement: suppl. [file NIHMS662495-supplement-suppl_.zip › BalasConlin_SyntheticTextures/208s.1_left.jpg]

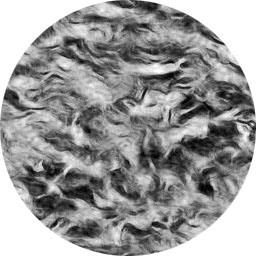

Supplement: suppl. [file NIHMS662495-supplement-suppl_.zip › BalasConlin_SyntheticTextures/208s.1_right.jpg]

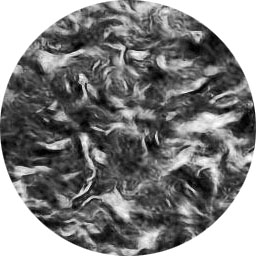

Supplement: suppl. [file NIHMS662495-supplement-suppl_.zip › BalasConlin_SyntheticTextures/208s.2_left.jpg]

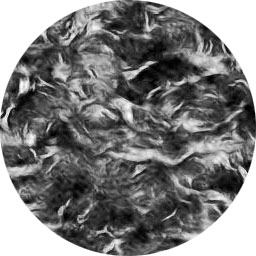

Supplement: suppl. [file NIHMS662495-supplement-suppl_.zip › BalasConlin_SyntheticTextures/208s.2_right.jpg]

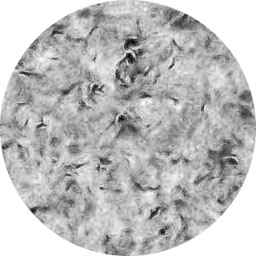

Supplement: suppl. [file NIHMS662495-supplement-suppl_.zip › BalasConlin_SyntheticTextures/228s.1_left.jpg]

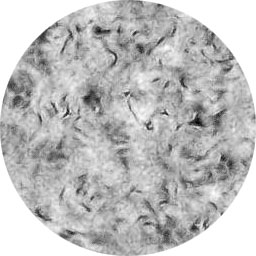

Supplement: suppl. [file NIHMS662495-supplement-suppl_.zip › BalasConlin_SyntheticTextures/228s.1_right.jpg]

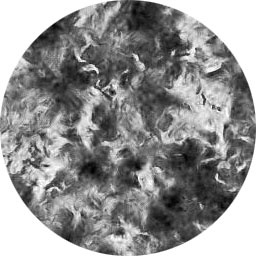

Supplement: suppl. [file NIHMS662495-supplement-suppl_.zip › BalasConlin_SyntheticTextures/228s.2_left.jpg]

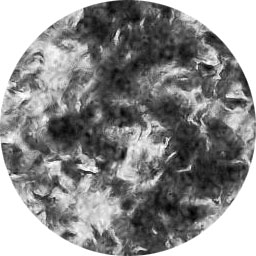

Supplement: suppl. [file NIHMS662495-supplement-suppl_.zip › BalasConlin_SyntheticTextures/228s.2_right.jpg]

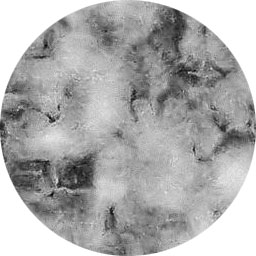

Supplement: suppl. [file NIHMS662495-supplement-suppl_.zip › BalasConlin_SyntheticTextures/231s.1_left.jpg]

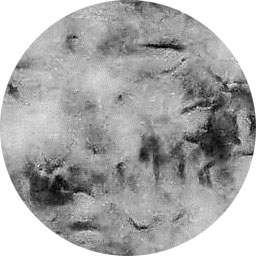

Supplement: suppl. [file NIHMS662495-supplement-suppl_.zip › BalasConlin_SyntheticTextures/231s.1_right.jpg]

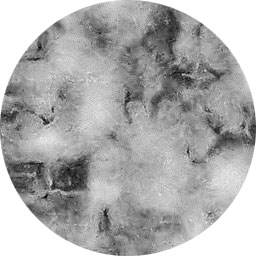

Supplement: suppl. [file NIHMS662495-supplement-suppl_.zip › BalasConlin_SyntheticTextures/231s.2_left.jpg]

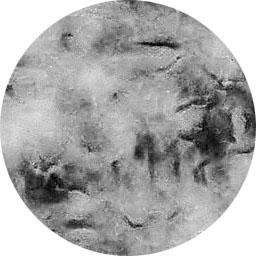

Supplement: suppl. [file NIHMS662495-supplement-suppl_.zip › BalasConlin_SyntheticTextures/231s.2_right.jpg]

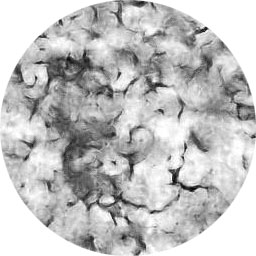

Supplement: suppl. [file NIHMS662495-supplement-suppl_.zip › BalasConlin_SyntheticTextures/236s.1_left.jpg]

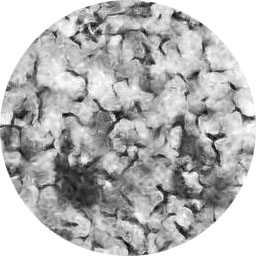

Supplement: suppl. [file NIHMS662495-supplement-suppl_.zip › BalasConlin_SyntheticTextures/236s.1_right.jpg]

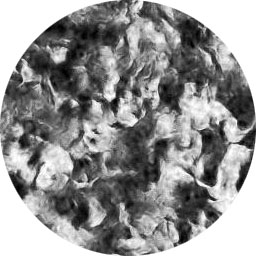

Supplement: suppl. [file NIHMS662495-supplement-suppl_.zip › BalasConlin_SyntheticTextures/236s.2_left.jpg]

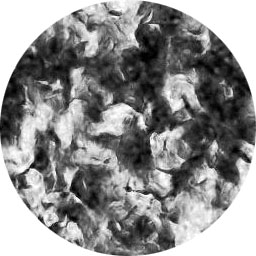

Supplement: suppl. [file NIHMS662495-supplement-suppl_.zip › BalasConlin_SyntheticTextures/236s.2_right.jpg]

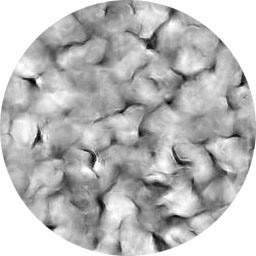

Supplement: suppl. [file NIHMS662495-supplement-suppl_.zip › BalasConlin_SyntheticTextures/250s.1_left.jpg]

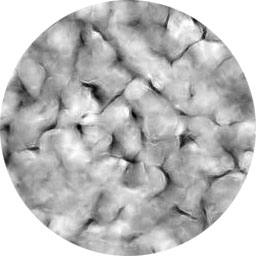

Supplement: suppl. [file NIHMS662495-supplement-suppl_.zip › BalasConlin_SyntheticTextures/250s.1_right.jpg]

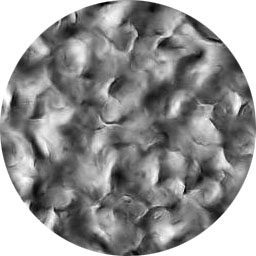

Supplement: suppl. [file NIHMS662495-supplement-suppl_.zip › BalasConlin_SyntheticTextures/250s.2_left.jpg]

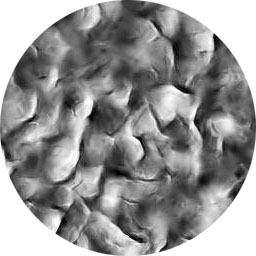

Supplement: suppl. [file NIHMS662495-supplement-suppl_.zip › BalasConlin_SyntheticTextures/250s.2_right.jpg]

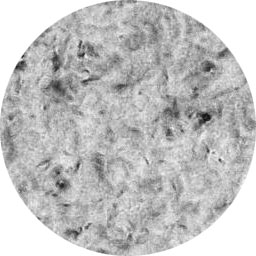

Supplement: suppl. [file NIHMS662495-supplement-suppl_.zip › BalasConlin_SyntheticTextures/25s.1_left.jpg]

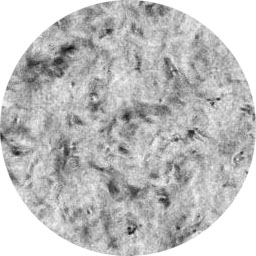

Supplement: suppl. [file NIHMS662495-supplement-suppl_.zip › BalasConlin_SyntheticTextures/25s.1_right.jpg]

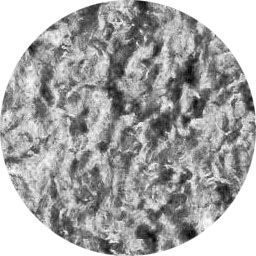

Supplement: suppl. [file NIHMS662495-supplement-suppl_.zip › BalasConlin_SyntheticTextures/25s.2_left.jpg]

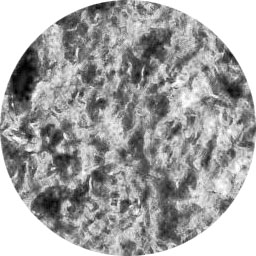

Supplement: suppl. [file NIHMS662495-supplement-suppl_.zip › BalasConlin_SyntheticTextures/25s.2_right.jpg]

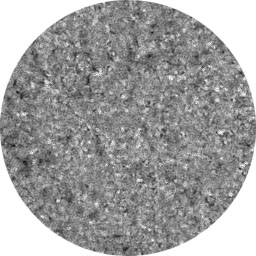

Supplement: suppl. [file NIHMS662495-supplement-suppl_.zip › BalasConlin_SyntheticTextures/33s.1_left.jpg]

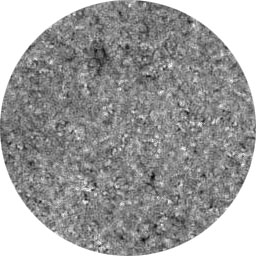

Supplement: suppl. [file NIHMS662495-supplement-suppl_.zip › BalasConlin_SyntheticTextures/33s.1_right.jpg]

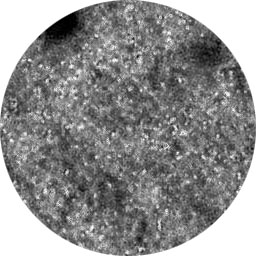

Supplement: suppl. [file NIHMS662495-supplement-suppl_.zip › BalasConlin_SyntheticTextures/33s.2_left.jpg]

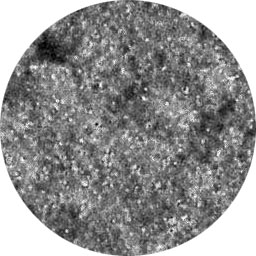

Supplement: suppl. [file NIHMS662495-supplement-suppl_.zip › BalasConlin_SyntheticTextures/33s.2_right.jpg]

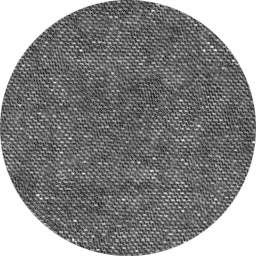

Supplement: suppl. [file NIHMS662495-supplement-suppl_.zip › BalasConlin_SyntheticTextures/43s.1_left.jpg]

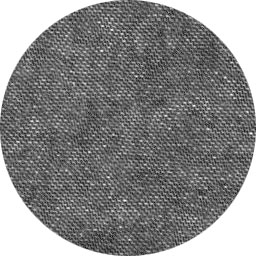

Supplement: suppl. [file NIHMS662495-supplement-suppl_.zip › BalasConlin_SyntheticTextures/43s.1_right.jpg]

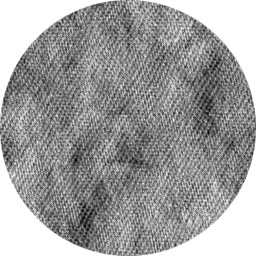

Supplement: suppl. [file NIHMS662495-supplement-suppl_.zip › BalasConlin_SyntheticTextures/43s.2_left.jpg]

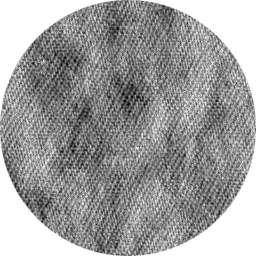

Supplement: suppl. [file NIHMS662495-supplement-suppl_.zip › BalasConlin_SyntheticTextures/43s.2_right.jpg]

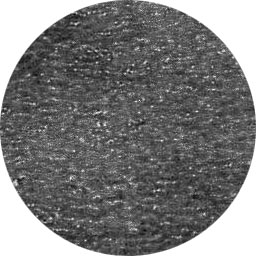

Supplement: suppl. [file NIHMS662495-supplement-suppl_.zip › BalasConlin_SyntheticTextures/44s.1_left.jpg]

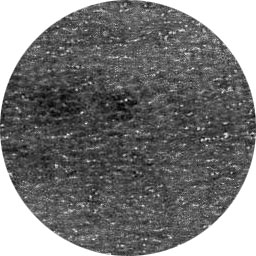

Supplement: suppl. [file NIHMS662495-supplement-suppl_.zip › BalasConlin_SyntheticTextures/44s.1_right.jpg]

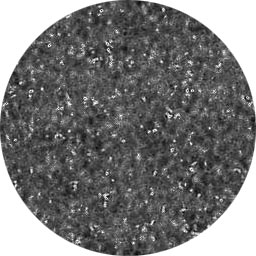

Supplement: suppl. [file NIHMS662495-supplement-suppl_.zip › BalasConlin_SyntheticTextures/44s.2_left.jpg]

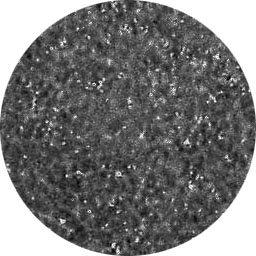

Supplement: suppl. [file NIHMS662495-supplement-suppl_.zip › BalasConlin_SyntheticTextures/44s.2_right.jpg]

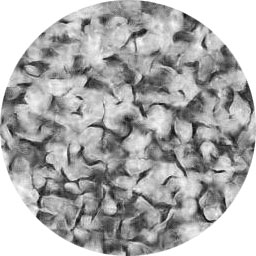

Supplement: suppl. [file NIHMS662495-supplement-suppl_.zip › BalasConlin_SyntheticTextures/49s.1_left.jpg]

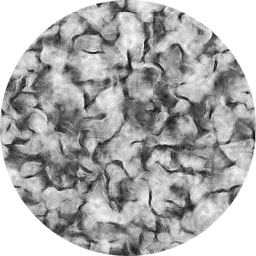

Supplement: suppl. [file NIHMS662495-supplement-suppl_.zip › BalasConlin_SyntheticTextures/49s.1_right.jpg]

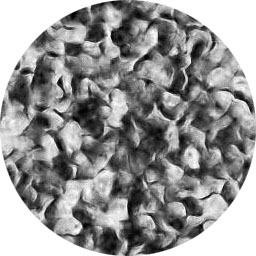

Supplement: suppl. [file NIHMS662495-supplement-suppl_.zip › BalasConlin_SyntheticTextures/49s.2_left.jpg]

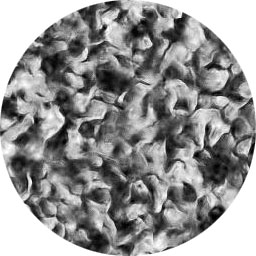

Supplement: suppl. [file NIHMS662495-supplement-suppl_.zip › BalasConlin_SyntheticTextures/49s.2_right.jpg]

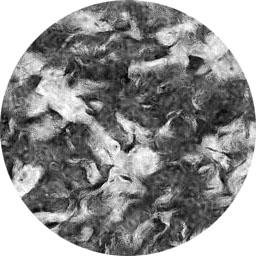

Supplement: suppl. [file NIHMS662495-supplement-suppl_.zip › BalasConlin_SyntheticTextures/53s.1_left.jpg]

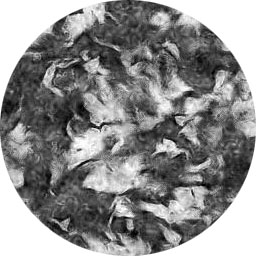

Supplement: suppl. [file NIHMS662495-supplement-suppl_.zip › BalasConlin_SyntheticTextures/53s.1_right.jpg]

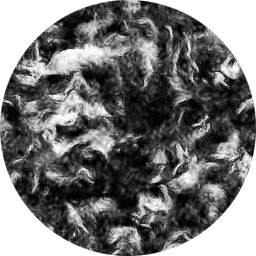

Supplement: suppl. [file NIHMS662495-supplement-suppl_.zip › BalasConlin_SyntheticTextures/53s.2_left.jpg]

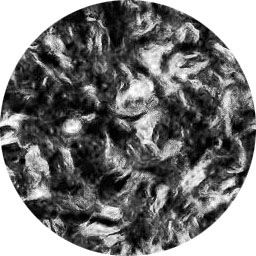

Supplement: suppl. [file NIHMS662495-supplement-suppl_.zip › BalasConlin_SyntheticTextures/53s.2_right.jpg]
